# Generating Clinical-Grade Gene–Disease Validity Classifications Through the ClinGen Data Platforms

**DOI:** 10.1146/annurev-biodatasci-102423-112456

**Published:** 2024-07-24

**Authors:** Matt W. Wright, Courtney L. Thaxton, Tristan Nelson, Marina T. DiStefano, Juliann M. Savatt, Matthew H. Brush, Gloria Cheung, Mark E. Mandell, Bryan Wulf, TJ Ward, Scott Goehringer, Terry O’Neill, Phil Weller, Christine G. Preston, Ingrid M. Keseler, Jennifer L. Goldstein, Natasha T. Strande, Jennifer McGlaughon, Danielle R. Azzariti, Ineke Cordova, Hannah Dziadzio, Lawrence Babb, Kevin Riehle, Aleksandar Milosavljevic, Christa Lese Martin, Heidi L. Rehm, Sharon E. Plon, Jonathan S. Berg, Erin R. Riggs, Teri E. Klein

**Affiliations:** 1 Department of Biomedical Data Science, Stanford University School of Medicine, Stanford, California, USA; 2 Department of Genetics, University of North Carolina, Chapel Hill, North Carolina, USA; 3 Geisinger, Danville, Pennsylvania, USA; 4 Medical and Population Genetics, Broad Institute of MIT and Harvard, Cambridge, Massachusetts, USA; 5 Department of Biomedical Informatics, University of Colorado Anschutz Medical Campus, Aurora, Colorado, USA; 6 Department of Molecular and Human Genetics, Baylor College of Medicine, Houston, Texas, USA; 7 Department of Pediatrics, Division of Hematology-Oncology, Baylor College of Medicine, Houston, Texas, USA; 8 Departments of Medicine (Biomedical Informatics Research) and Genetics, Stanford University School of Medicine, Stanford, California, USA

**Keywords:** biocuration, precision medicine, clinical genetics, data standards, research informatics, data harmonization

## Abstract

Clinical genetic laboratories must have access to clinically validated biomedical data for precision medicine. A lack of accessibility, normalized structure, and consistency in evaluation complicates interpretation of disease causality, resulting in confusion in assessing the clinical validity of genes and genetic variants for diagnosis. A key goal of the Clinical Genome Resource (ClinGen) is to fill the knowledge gap concerning the strength of evidence supporting the role of a gene in a monogenic disease, which is achieved through a process known as Gene–Disease Validity curation. Here we review the work of ClinGen in developing a curation infrastructure that supports the standardization, harmonization, and dissemination of Gene–Disease Validity data through the creation of frameworks and the utilization of common data standards. This infrastructure is based on several applications, including the ClinGen GeneTracker, Gene Curation Interface, Data Exchange, GeneGraph, and website.

## INTRODUCTION

**Figure 1:**
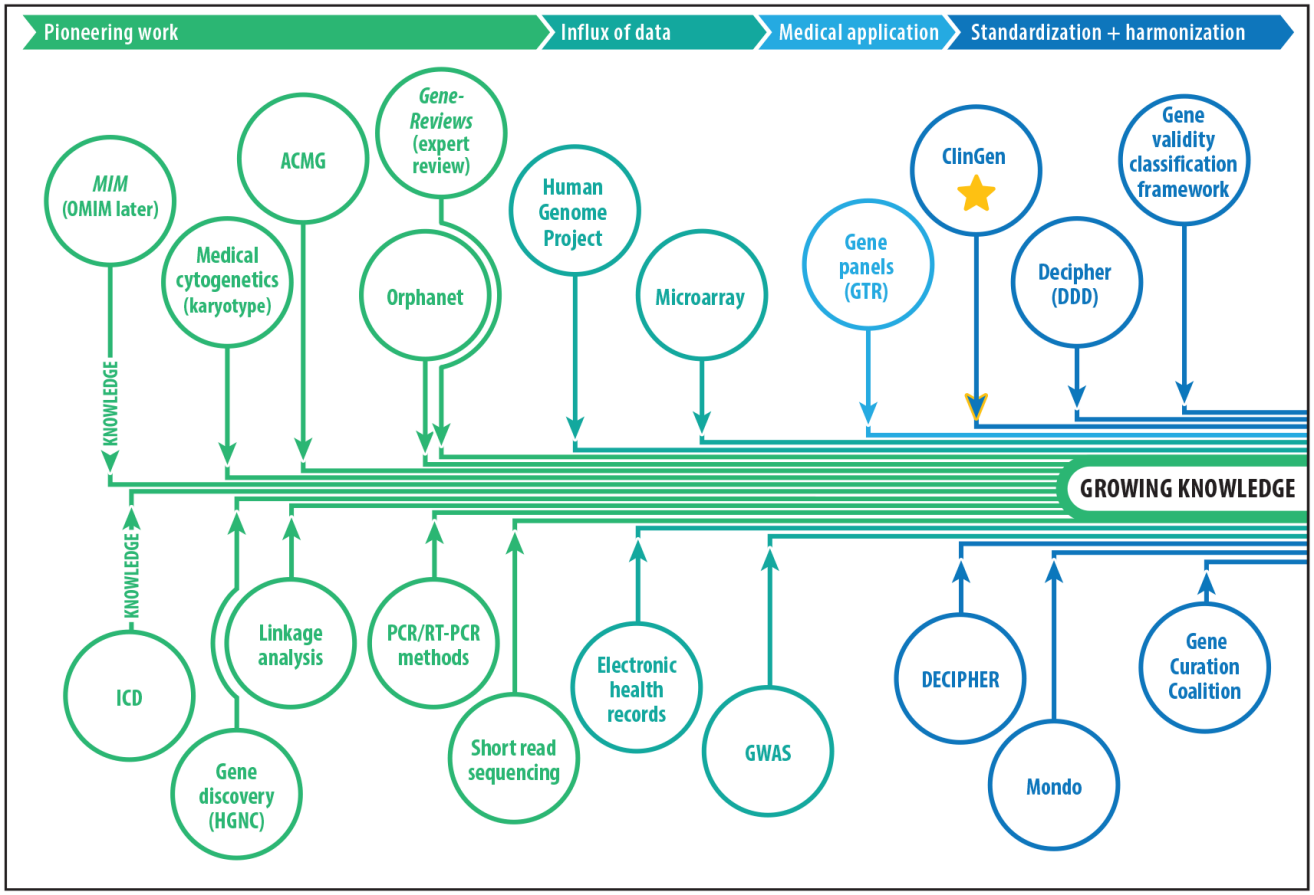
Increasing genomic data influx due to advancements in knowledge bases and technologies. Over time the development of several knowledge bases (e.g., OMIM, Mondo, *GeneReviews*) and/or technologies (e.g., polymerase chain reaction, microarray, gene panels) aimed toward evaluating and discovering genes associated with disease resulted in an ever-increasing amount of data. With this growing knowledge, ClinGen was launched in order to develop strategies to evaluate the clinical validity of gene–disease relationships and sort through much of the data that were generated prior to its establishment. This image represents a small fraction of the genomic knowledge bases and technologies that contributed to the influx of data. Abbreviations: ACMG, American College of Medical Genetics and Genomics; ClinGen, Clinical Genome Resource; DDD, Deciphering Developmental Disorders; GTR, Genetic Testing Registry; GWAS, genome-wide association study; HGNC, Human Genome Organization Gene Nomenclature Committee; ICD, International Classification of Diseases; *MIM*, *Mendelian Inheritance in Man*; Mondo, Monarch Disease Ontology; OMIM, Online Mendelian Inheritance in Man; PCR, polymerase chain reaction; RT-PCR, reverse transcription polymerase chain reaction.

For over half a century, researchers have made efforts to develop and maintain inventories that assess and catalog genes involved in human disease. These efforts began in 1966 with the seminal work of Victor McKusick in developing the journal *Mendelian Inheritance in Man* (*MIM*) (1). Today, known as Online Mendelian Inheritance in Man (OMIM) due to its web-based presence, it continues to be a resource of curated data of genes implicated in human disease (Figure 1). Several additional resources [e.g., the Human Genome Organization Gene Nomenclature Committee (HGNC) (2), Orphanet (https://www.orpha.net), *GeneReviews* (https://www.ncbi.nlm.nih.gov/books/NBK1116/), etc.] aimed at categorizing, describing, and cataloging the clinical relevance of genes have since emerged, building from *MIM*’s initial efforts, while concomitant technological advancements (e.g., Sanger sequencing, linkage analysis, microarray, etc.) have fueled gene discovery. A pinnacle moment in clinical genetics occurred in 2003 when the Human Genome Project (HGP) produced a nearly complete sequence of the human genome, thus accelerating gene identification, especially for rare, monogenic disorders (3). Simultaneously, the HGP contributed to significant advances in sequencing technologies and applications (e.g., gene panels, exome sequencing, etc.), resulting in a burgeoning accumulation of human genetic data that amounted to an estimated 30 million human genomic sequences generated by 2021 (4). The increase and growing availability of genomic data led to dramatic improvements in comparative analyses of genotype/phenotype correlations and the interpretation of disease causality of genes and genomic variants. It also contributed to a growing number of conflicting and disparate interpretations brought about by siloed data and analyses and a general lack and inconsistent use of standards and frameworks within and between those performing the interpretations. Such conflicts could have significant impacts on patient diagnosis, care, and treatment. Further, while OMIM and other resources cataloged the published assertions of genes involved in disease, the strength of these assertions and their validity were often unknown and based on few publications, leaving a fundamental gap in the knowledge repositories that could help to provide better medical insight to those individuals with a monogenic disease.

Given the general landscape and increasing number of assertions of genes and variants involved in human disease, the Clinical Genome Resource (ClinGen) was established in 2013 to create an authoritative central resource to define the clinical relevance of genes and variants for use in precision medicine and research (5). ClinGen sought to fill in the missing link and build on prior resources by developing frameworks to assess the strength of evidence of genes and genomic variants in disease. One of the key goals included developing infrastructure and standards to assist in the aggregation, annotation, and evaluation of relevant data in order to establish the strength of evidence that a gene is involved in a human disease, called Gene–Disease Validity (6).

The development and continued refining of a semiquantitative scoring metric by the ClinGen Gene Curation Working Group (GCWG) provide one of the first publicly available and consistent approaches for the evaluation of genes in monogenic diseases (7). The ClinGen Gene–Disease Validity curation process expands on other resources, such as OMIM and Orphanet, by providing a classification scheme, supported by a semiquantitative metric, indicating the strength of gene–disease relationships, thus leveling the assertions made in the literature, which often are inherently biased toward novelty. The additional information provided by the ClinGen Gene–Disease Validity process complements these resources by adding to the growing knowledge base and understanding, while also providing an essential validation aspect that other resources do not include. The Gene–Disease Validity process, including the curation, classification, validation, and approval of gene–disease relationships, is performed by a group of clinical domain experts termed a Gene Curation Expert Panel (GCEP). The importance of the Gene–Disease Validity process and its utility to the clinical and diagnostic community are exemplified in recommendations published by the American College of Medical Genetics and Genomics on the use of the ClinGen Gene–Disease Validity framework and classifications when considering genes to include on, and/or report as part of, diagnostic testing panels (8).

As part of the Gene–Disease Validity assessment standards, ClinGen promotes, and often requires, the use of standard ontologies and stable identifiers to account for the ever-evolving changes to clinical nomenclature; these include the Human Phenotype Ontology (HPO) (9), Monarch Disease Ontology (Mondo) (10), and OMIM. Through collaborative efforts with these and other pioneering and important resources, ClinGen continues to harmonize its data across different entities to encourage the consistency, transparency, and standardization of evaluated data for clinically relevant genes. To enable the necessary elements required of an authoritative resource, including standardized data collection, data evaluation, and data display, ClinGen implemented systems, applications, and data models for the collection, structuring, and standardization of annotated data. In the following sections we review the systems supporting the Gene–Disease Validity curation process and workflow, including defining the ClinGen curated monogenic disease entity (precuration), applying the semiquantitative metric (curation), and modeling the data in support of the publication of the Gene–Disease Validity classifications and relevant data to the ClinGen website (https://clinicalgenome.org) for user consumption (dissemination).

## PRECURATION AND GENETRACKER

The goal of the Gene–Disease Validity curation process is to assess the strength of evidence supporting a gene’s role in a monogenic disease. At first glance this may seem relatively straight-forward; however, the process of defining the curated disease entity is complicated by the fact that ~30% of asserted monogenic disease genes are associated with two or more phenotypes according to OMIM (https://www.omim.org/statistics/geneMap). For example, for genes such as *LMNA*, *FBN1*, and *ABCA4* that are associated with 11, 8, and 6 phenotypes per OMIM (as of September 2023), respectively, it is crucial to assess whether some or all of the asserted phenotypes are part of a phenotypic spectrum (and therefore should be lumped) or whether any represent a distinct entity(ies) (and therefore should be split). This determination is important, because all ClinGen Gene–Disease Validity classifications depend on the inclusion and/or exclusion of relevant data.

The ClinGen Lumping and Splitting Working Group (LSWG) was convened to establish recommendations to aid biocurators and experts in defining the curated monogenic disease entity (11). As part of this effort, the LSWG developed recommendations supported by a high-level process to collect information on gene–disease assertions, termed precuration. Precuration includes a high-level review of the current knowledge pertaining to the assertions, mechanism(s), phenotypic variability, and inheritance pattern across the reported disease entities for any given gene. To assist in the structuring of the precuration data to allow for findable, accessible, interoperable, and reusable (FAIR) principles (12) of data transparency, ClinGen developed a web-based application called GeneTracker that catalogs the lumping and splitting criteria and precuration data of all Gene–Disease Validity classifications across all GCEPs. The result of the precuration process is a gene–disease–mode of inheritance (GDM) entity captured in GeneTracker. For genes asserted to be involved in more than one disease, the precuration process supports the distinction of the asserted phenotypes, represented by OMIM phenotypes (MIM number), with the final monogenic disease entity, represented using a Mondo identifier. Thus, the precuration process effectively links OMIM and Mondo identifiers, allowing for collaboration and harmonization of data between these resources.

**Figure 2:**
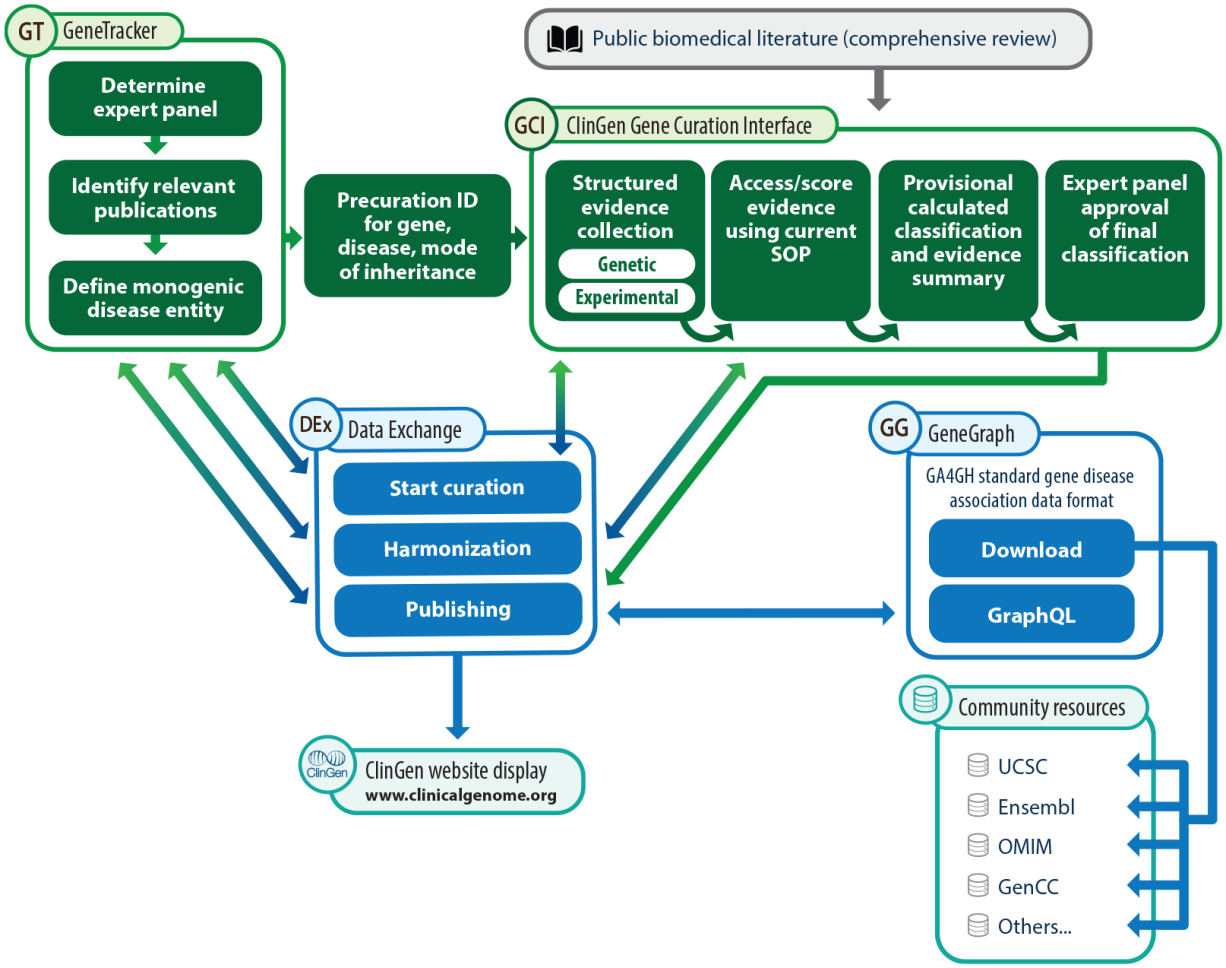
ClinGen’s Gene–Disease Validity curation workflow and supporting infrastructure. During the course of curation, publication, and dissemination, a ClinGen Gene–Disease Validity curation passes through multiple systems. A curation is initiated in GeneTracker, where the appropriate gene, disease, and mode of inheritance; the correct expert panel to perform the curation; and an initial review of the relevant literature are recorded. This information is passed to the ClinGen GCI, where details about the evidence are recorded, structured, and scored according to the current SOP, based on a comprehensive review of the literature. This work is reviewed according to the policies of the expert panel performing the work, and a final classification is agreed upon and approved in the GCI. Following approval the curation is sent to GeneGraph via the ClinGen Data Exchange, an Apache Kafka–based messaging system, which facilitates durable and auditable transfers of data between systems. In GeneGraph the data received are transformed from the format used to support the user interface of the GCI into a structured, normalized format based on the Scientific Evidence and Provenance Information Ontology model. A queryable application programming interface based on GraphQL reflecting this model is presented to the ClinGen website, where curation data are viewable by the public. Currently summary curation data are available for download via the website; a goal is to present full, structured, computable data for download by resources in the community. Abbreviations: GA4GH, Global Alliance for Genomics and Health; GCI, Gene Curation Interface; GenCC, Gene Curation Coalition; OMIM, Online Mendelian Inheritance in Man; SOP, standard operating procedure; UCSC, University of California Santa Cruz Genome Browser.

GeneTracker facilitates linking of the precuration record, which establishes the gene–disease relationship, with the full Gene–Disease Validity curation and classification by issuing a unique precuration identifier (precuration ID). Given the importance of precuration, the precuration ID became a required element in 2023 to initiate a gene–disease record in the Gene Curation Interface (GCI), described below (Figure 2). All precuration data are published to the ClinGen website upon final approval of a Gene–Disease Validity classification. In addition to its role in cataloging the precuration criteria, GeneTracker is also an internal system of record to track all genes of interest for curation across all ClinGen GCEPs. Tracking GCEP gene lists facilitates reducing the redundancy of curation efforts and enhances communication between groups about the proposed classifications, thereby improving the efficiency of the Gene–Disease Validity curation process.

## GENE CURATION INTERFACE

Biocurators perform an indispensable role in the Gene–Disease Validity curation process by ensuring that pertinent biomedical data adhere to FAIR guiding principles (12). These FAIR principles essentially describe the beneficial work of biocurators: collecting unstructured data, confirming and corroborating the collected data, structuring them using commonly accepted standards, and then storing them in formats that make them easily accessible. Biocuration provides context to data; providing meaningful descriptors, adding unique identifiers, and linking these elements to pertinent metadata vastly improve the utility of the data. The GCI was developed as a platform to support ClinGen GCEP biocurators in the comprehensive annotation and assessment of the Gene–Disease Validity curation process. The GCI programmatically guides biocurators through the structured and standardized data collection process, as well as application of the Gene–Disease Validity guidelines, in a controlled workflow to enforce the rigor, quality, and transparency of the final Gene–Disease Validity classification.

The GCI was developed via a user-led design process, with the software development teams working alongside a subgroup of the ClinGen GCWG (7). The GCI continues to evolve with the input of core members of the GCWG, GCEPs, and the ClinGen gene curation community (https://www.clinicalgenome.org/working-groups/gene-curation/). The Gene–Disease Validity standard operating procedure (SOP) (https://www.clinicalgenome.org/curation-activities/gene-disease-validity/training-materials/) provides detailed best practice recommendations for establishing gene–disease clinical validity, while the GCI enables consistent application of the semiquantitative framework. Synchronization between the GCI and its capabilities and the Gene–Disease Validity SOP is paramount to the curation process, and flexibility is needed to allow for updates and changes with time. The software for the GCI is freely and openly available in perpetuity via publicly accessible web pages and a publicly available GitHub repository for the codebase (https://github.com/ClinGen/gene-and-variant-curation-tools). The GCI website (https://curation.clinicalgenome.org) allows access to a common interface for both the GCI and the related Variant Curation Interface (VCI), which is a previously described comprehensive germline variant classification platform (13). A shared platform between the GCI and the VCI allows for interoperability and data sharing between the two systems, which enhance efficiency, accessibility, and user experience. User access is via authenticated login, which is required in order to document the provenance of evidence added to both curation interfaces. Gene–Disease Validity curation in the GCI is restricted to ClinGen-approved GCEP members to ensure that all resulting classifications have been meticulously generated through application of ClinGen’s Gene–Disease Validity SOP. It is important to have this level of rigor in order to produce a clinical-grade output for use in precision medicine.

## GENE–DISEASE VALIDITY CURATION WORKFLOW SUPPORTED BY THE GENE CURATION INTERFACE

The GCI supports biocurators in structuring, evaluating, and classifying the data according to the current Gene–Disease Validity curation workflow (Figure 2). Details of each workflow step are elaborated below.

### Starting a Gene–Disease Validity Curation

The primary unit that is curated in the GCI is the GDM. Each GCI record is defined by a GDM triad, which curators recognize as the primary information from which the connection between any given gene and disease is asserted. Prior to creating a new GDM in the GCI, a precuration ID must be obtained by completing precuration in GeneTracker, as described above. The HGNC identifier is linked to the record, along with the current HGNC-approved gene symbol, to account for possible nomenclature updates over time (2). The disease entity is defined using a Mondo identifier (10), and the mode of inheritance (MOI) is selected from a list provided by the HPO (9). These three elements must be consistently recorded between the precuration record in GeneTracker and the GCI to start a record.

Upon creation, each GDM is given a permanent universally unique identifier (UUID) (14) to allow it to be tracked in perpetuity. This same UUID is used for tracking each GDM classification across the Gene–Disease Validity curation infrastructure, facilitating the linking of data for GDMs across multiple platforms, including GeneTracker and the ClinGen website. The GCI allows GCEPs to curate under a single entity, known as an affiliation. Each GCEP represents a single affiliation, and all of its members are able to access and contribute to GDMs contained within their affiliation. Only one GDM can exist in the GCI for each unique gene–disease–MOI triad, and each one is solely curated by one specific GCEP at a time. However, a group that collaborates on a GDM can be given an attribution as a secondary contributor to acknowledge the efforts of multiple GCEPs on the same record. Further, GDMs can be transferred to another GCEP as necessary, while the architecture of the UUID and GCI allows for historical records of a GDM to be retained for auditing. This information is harmonized across other gene curation–related systems in ClinGen (e.g., GeneTracker).

### Structured Evidence Collection and Scoring

Following the initiation of a GDM curation record, supporting and/or conflicting evidence for the gene–disease relationship must be collected to apply the semiquantitative metric. The GCI supports the structured, unambiguous addition of evidence sources by requiring the use of identifiers. Wherever possible, controlled vocabularies and ontologies are required, thus promoting consistency in how concepts are defined and supporting cross-referencing and searching of the final classifications. A full list of ontologies and structured vocabularies implemented to promote consistency in data collection and use across the ClinGen Gene–Disease Validity curation infrastructure is provided in Table 1.

The majority of evidence for Gene–Disease Validity classifications comes from published literature; therefore, PubMed identifiers (PMIDs) are the primary evidence identifier associated with curations. Recent movements in the scientific community have encouraged and outlined the benefits of prepublishing data to preprint servers (e.g., bioRxiv, medRxiv) or publicly available databases (e.g., ClinVar) (29), as this can circumvent lengthy journal reviews and expedite public access to data. Such early access also allows invested users outside of the usual peer-review process to view data and make suggestions for improvements (30). To be flexible and evolve with changing community recommendations and standards, the GCI is expanding the allowable primary identifiers for evidence to include digital object identifiers (DOIs) and ClinVar submission identifiers. DOIs also provide the ability to curate information from books, reviews, and other primary sources of literature that may not have PMIDs.

There are two main types of evidence for a GDM, (*a*) genetic evidence, which focuses on data observed in humans, and (*b*) experimental evidence, which focuses on data observed at the level of biochemical, cellular, and/or model system(s). Within each of these main evidence types, subcategories exist to stratify the data and provide additional details as appropriate for the application of the semiquantitative metric (7). For genetic evidence, the subcategories include case-level evidence, which describes groups, families, or individuals who harbor a variant(s) of the gene of interest, and case-control evidence, which describes statistical analyses used to evaluate whether or not single or multiple genetic variants within one gene are enriched in cases compared to control groups. The types of experimental evidence fall into four broad themes: (*a*) functional, (*b*) functional alteration, (*c*) model(s), and (*d*) rescue. Based on the selected type of evidence, the GCI presents users with evidence collection forms containing structured fields that utilize identifiers or controlled vocabularies to the extent possible (Table 1), as well as drop-down menus or validation modals to reduce error (e.g., typographical). Free text fields are also employed in areas in which controlled vocabularies and identifiers are not available or where a succinct review of rationale is important for context. The user interface has built-in logic in some areas to reduce discrepancies, increase efficiency, and enhance the user experience. For example, users have the ability to copy phenotypic descriptors (HPO identifier and/or free text) from a group to a family and/or individual case report to reduce the redundancy of identically relevant and valid evidence items across multiple linked resources.

Appropriate attribution of a genetic variant is paramount for the scoring of evidence. The Human Genome Variation Society (HGVS) provides a nomenclature schema for DNA sequence variants; however, as with other nomenclatures, HGVS variant nomenclature has changed with time due to improving resolution of the human genome. Nomenclature changes over time can present a challenge for the correct attribution of a genetic variant. To overcome this challenge, the GCI accepts three different forms of variant identifiers, a ClinVar Variation identifier, a ClinGen Allele Registry Canonical Allele identifier (CA ID), and/or a ClinGen Allele Registry Canonical Allele Copy Number identifier (CACN ID). The ClinGen Allele Registry identifiers provide an advantage over other variant identifiers (e.g., dbSNP) (31) because each variant is mapped across multiple genomic builds (i.e., NCBI36, GrCH37, etc.) and transcripts [e.g., the European Molecular Biology Laboratory European Bioinformatics Institute’s (EMBL-EBI’s) Ensembl transcripts and National Center for Biotechnology Information’s (NCBI’s) RefSeq transcripts] (32, 33); variants are also highlighted in the consensus Matched Annotation from NCBI and EMBL-EBI (MANE) Select transcript(s) (34), and CA IDs and CACN IDs are linked to ClinVar IDs, dbSNP, and more. The GCI pulls variant transcript-related data, such as all known HGVS terms, from the ClinGen Allele Registry to assist in the validation of the allele and the structured evidence collection.

Upon completing the required fields for an evidence type, a biocurator can score each individual piece of evidence, mark it as contradictory, or indicate it needs further review. If a score is indicated, a default calculated score that is evidence type–specific is provided. The default score can also be modified within a predefined range as governed by the Gene–Disease Validity SOP. Further, a score of zero can also be applied to indicate that the evidence was reviewed by the curator to be incomplete, not up to standards set by the Gene–Disease Validity SOP or GCEP, or in general not supportive of the gene’s role in disease. Any changes to a default score require the addition of a rationale for the decision, which is published in the final curation as a means to increase transparency of the decision-making and classification process.

### Calculated Classification Matrix and Curation Summary

**Figure 3:**
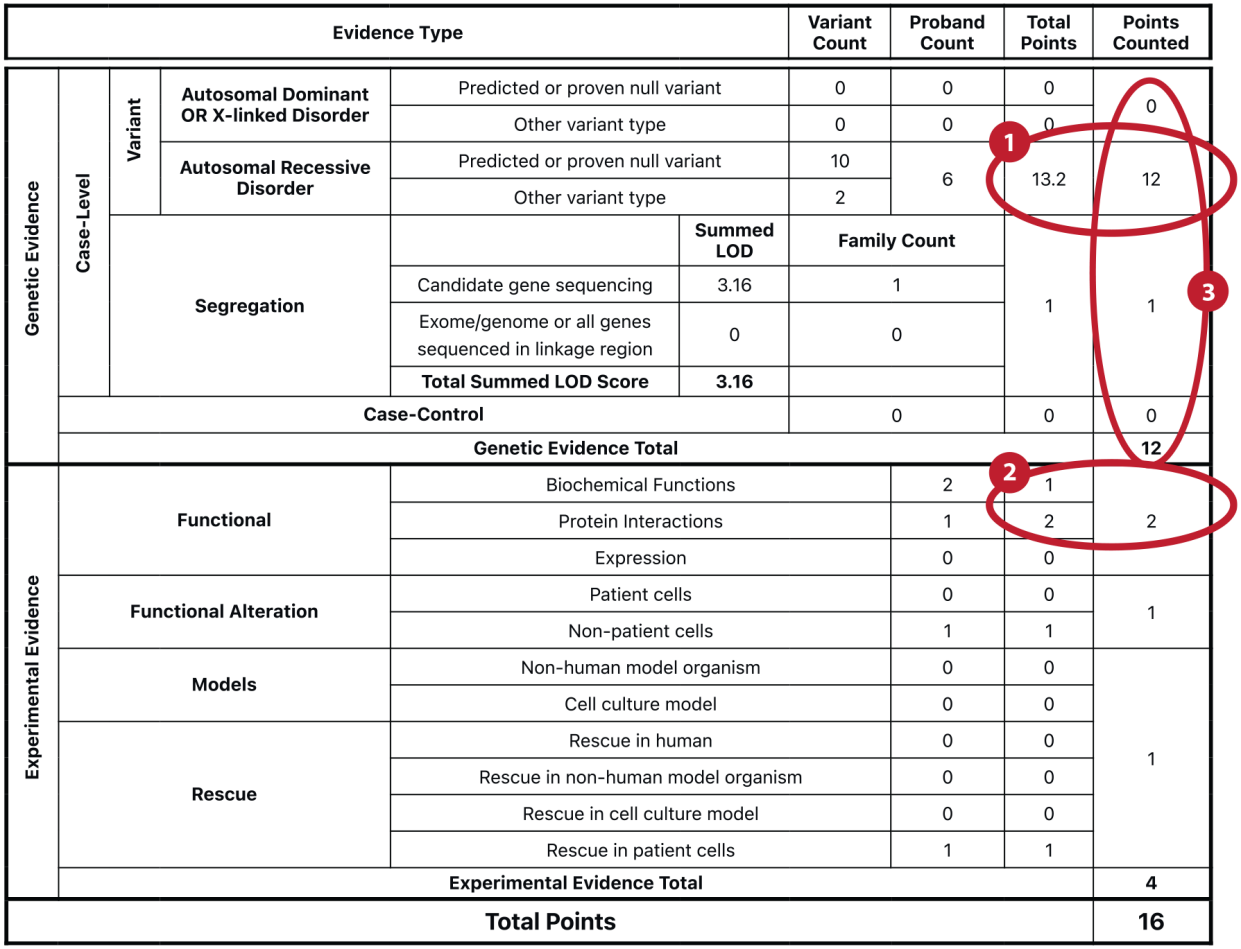
Example of a Calculated Classification Matrix in the Gene Curation Interface (GCI). This matrix shows the evidence scores automatically tabulated for the *PEX19* (HGNC:9713)/peroxisome biogenesis disorder (MONDO:0019234)/autosomal recessive inheritance (HP:0000007) gene–disease–mode of inheritance within the GCI, as curated by the ClinGen Peroxisomal Disorders Gene Curation Expert Panel (35). Points have been capped in three places: (①) For variant evidence the 13.2 total points have been capped to 12 points counted, (②) for functional evidence the 3 total points have been capped to 2 points counted, and (③) for all genetic evidence the 13 total points have been capped to 12 points counted. A version of this matrix is replicated in the final classification published on the ClinGen website: https://search.clinicalgenome.org/kb/genes/HGNC:9713. Abbreviation: LOD, logarithm of the odds.

As scores accumulate, the GCI aggregates and automatically generates a Calculated Classification Matrix that provides a synopsis of the curated evidence and the current point tally. This matrix can aid biocurators in determining if sufficient evidence and scores have been reached within the broad and subcategory types, and to allow them to see the current classification based on the score. The GCI automatically caps the total points for each broad evidence category (genetic and experimental) and subcategory (e.g., genetic–segregation, experimental–function) consistent with current Gene Disease–Validity SOP guidance. Capping ensures that individual evidence types are weighted appropriately in their contribution to the overall classification; without capping, an abundance of weak evidence from one category could raise the classification beyond what would be expected based on the evidence as a whole. The capped score is represented within the column labeled Points Counted on the Calculated Classification Matrix for each evidence type; the column labeled Total Points indicates all scores that were tallied. As an example, in Figure 3 the 13.2 total points collected for genetic variant evidence exceed the 12 maximum allowable points for that evidence type category; thus, the Points Counted column caps at 12 points. Similarly, the 3 total functional experimental evidence points collected across the combined categories are capped to 2 allowable points counted. The GCI thus automates capping of scored evidence and adjusts the final scores and suggested classification accordingly. A calculated classification is provided by the GCI based upon the total points in the classification matrix. A Limited classification indicates that at least 0.1 points have been calculated, Moderate indicates 7 to 11 points, Strong indicates 12 to 18 points with no replication over time, and Definitive indicates 12 to 18 total points plus evidence to indicate replication over time (i.e., two or more publications with convincing evidence over at least 3 years) (7). The experts can upgrade or downgrade a classification within one level of the calculated classification, provided that the rationale for this decision is documented in the GCI. Additionally, a No Known Disease Relationship classification can be applied if no evidence has been found in the literature to support a causal relationship between the gene of interest and the specified monogenic disease in humans; however, a subclassification of Animal Model Only can be applied to indicate that experimental evidence from a model organism suggests the gene–disease relationship.

Accompanying the Calculated Classification Matrix is a GCI Curation Summary table that details each individual piece of scored evidence for the current GDM (see Supplemental Figure 1 for an example of a complete Curation Summary table). The Curation Summary also highlights evidence marked as Review, as a reminder to discuss evidence details with the GCEP, while a Contradicts note is used to alert reviewers to consider whether any of the contradictory evidence is sufficient to consider the categories of Disputed or Refuted. All GDMs require a textual summary of the curation, termed the Evidence Summary. This summary provides an overview of all the evidence that was evaluated and used in creating the Gene–Disease Validity classification, as well as any other information necessary to understand the gene–disease relationship (e.g., common phenotypic features). The GCI provides a free text field for the Evidence Summary that is equipped to handle markup of text (e.g., italics, bolding, and paragraph breaks) so information can be conveyed with appropriate nomenclature standards and emphasis. A structured framework is provided for curators to emulate when constructing their Evidence Summaries.

### Provisional and Approved Classifications

To support expert panel review of the collected and structured data, the GCI generates a provisional classification. This classification represents a saved snapshot of the current curation that contains all of the data entered and/or scored by the biocurator to facilitate the review of evidence by the expert panel. This provisional status provides GCEPs with the ability to change or update data, evidence items, and/or scores. Recording statuses is important for tracking provenance and for comparative analysis of scoring. Further, the snapshot provides a historical view of curation changes over time and serves as a backup should any issues arise with the GCI that would result in information loss. Once a GCEP reviews and approves the data, any scoring changes can be reflected, which will result in a new provisional snapshot, followed by the ability to systematically capture additional attributes to the final approved curation, including the approval date and any other contributing ClinGen groups. This approval creates a new, unique snapshot in the GCI, which at this point allows a biocurator to proceed with publication of the final gene–disease relationship curation, including all evidence, classification, approval date, and approving GCEP(s), to the ClinGen website. Publication of the final approved GDM curation and classification to the ClinGen website is initiated in the GCI user interface, which automatically sends the full suite of knowledge, including comprehensive evidence evaluations and summaries, for immediate public display on the ClinGen website.

## GENE–DISEASE VALIDITY DISSEMINATION

To facilitate communication between internal systems and tools, ClinGen deployed an Apache Kafka–based system called the ClinGen Data Exchange (DEx). Within the context of Gene–Disease Validity curation, this system supports the dissemination and publication of the precurations, Gene–Disease Validity classifications, and accompanying curated data to the ClinGen website. The data from the GCI are delivered to a system developed by ClinGen called GeneGraph, which standardizes the data of the full suite of genomic knowledge curated for Gene–Disease Validity in a way that is consistent with ClinGen’s other curation products. GeneGraph takes the data output from the GCI, which is not directly suitable for query, publication, or downstream dissemination to other systems or tools, and transforms it into a form that can be directly queried and integrated with compatible datasets. GeneGraph stores these data in Apache Jena (https://jena.apache.org) and presents it to downstream users using a GraphQL (https://graphql.org) application programing interface (API). The ClinGen website queries the data available in GeneGraph using this API and presents a user interface over these data to the public.

### Data Model

The development of a consistent data model was imperative for the Gene–Disease Validity data, both to support internal ClinGen products and to present a cohesive view of data to external users in the community. At the core, most ClinGen classifications assess the level of evidence in support of a proposition. In the case of Gene–Disease Validity curation, this proposition is that variants that affect a specific gene’s function can be causative of a particular disease. The totality of evidence will be assessed along the scale from supporting to refuting this proposition. Similarly, ClinGen’s variant pathogenicity classifications are based around the proposition that a specific variant is causative of a disease; the available evidence may support this proposition (pathogenic variants), may refute it (benign variants), or may simply be inconclusive (variants of uncertain significance).

In order to ensure that ClinGen data are internally consistent and to support a unified presentation of the data on the ClinGen website, we applied the data model from the Scientific Evidence and Provenance Information Ontology (SEPIO) Framework (26) to all of the ClinGen classifications. This model fits the structure of our classifications and has additionally served as the foundation for the Global Alliance for Genomics and Health (GA4GH) emerging Variant Annotation (VA) Genomic Knowledge Standards (GKS), aligning with our goal to develop and support standards for data representation along with other groups with overlapping objectives. By connecting the standard terminologies using a standard data model, we open many possibilities to downstream users of the data, including their use in knowledge graphs for machine learning applications. GeneGraph presents a GraphQL API (described below), which integrates Gene–Disease Validity classifications with the other ClinGen curation data presented on the ClinGen website.

### GraphQL API

Given the depth and complexity of the structured data used for Gene–Disease Validity as well as the data offered by the other curation activities in ClinGen, GraphQL was selected as the standard for our API. A traditional representational state transfer (REST)-based API offers users and API designers less flexibility in situations where they need more dynamic options for the results they require, either (*a*) providing less data than desired in end points presenting many records and possibly forcing consumers to request detailed records individually, which can significantly increase delays and load on servers, or (*b*) returning a bigger set of data than an individual consumer needs, with the same deleterious effects. This inflexibility is particularly acute for the Gene–Disease Validity data, as there are over 100,000 individual evidence items and scores in the current dataset (and growing daily), with each evidence item having several additional descriptive data elements. In contrast to a traditional REST API, GraphQL allows a user to query for summary data over a large number of classifications, or for detailed information over a smaller number, with many gradations in between. Rather than offering a fixed set of fields when requesting a resource, GraphQL allows users of the API to specify what fields they want to retrieve as a part of the request, which may be deeply nested or very shallow, or any gradation in between. The framework offered by SEPIO makes it possible to support evolutions in the scoring structure for assessment without requiring significant (or any) changes to the underlying GraphQL schema; the pairing of GraphQL and the SEPIO framework makes for an approach that is flexible and maintainable. We currently have a version of this API in use internally, to be released to the public following internal validation and testing.

### ClinGen Data Exchange

**Figure 4:**
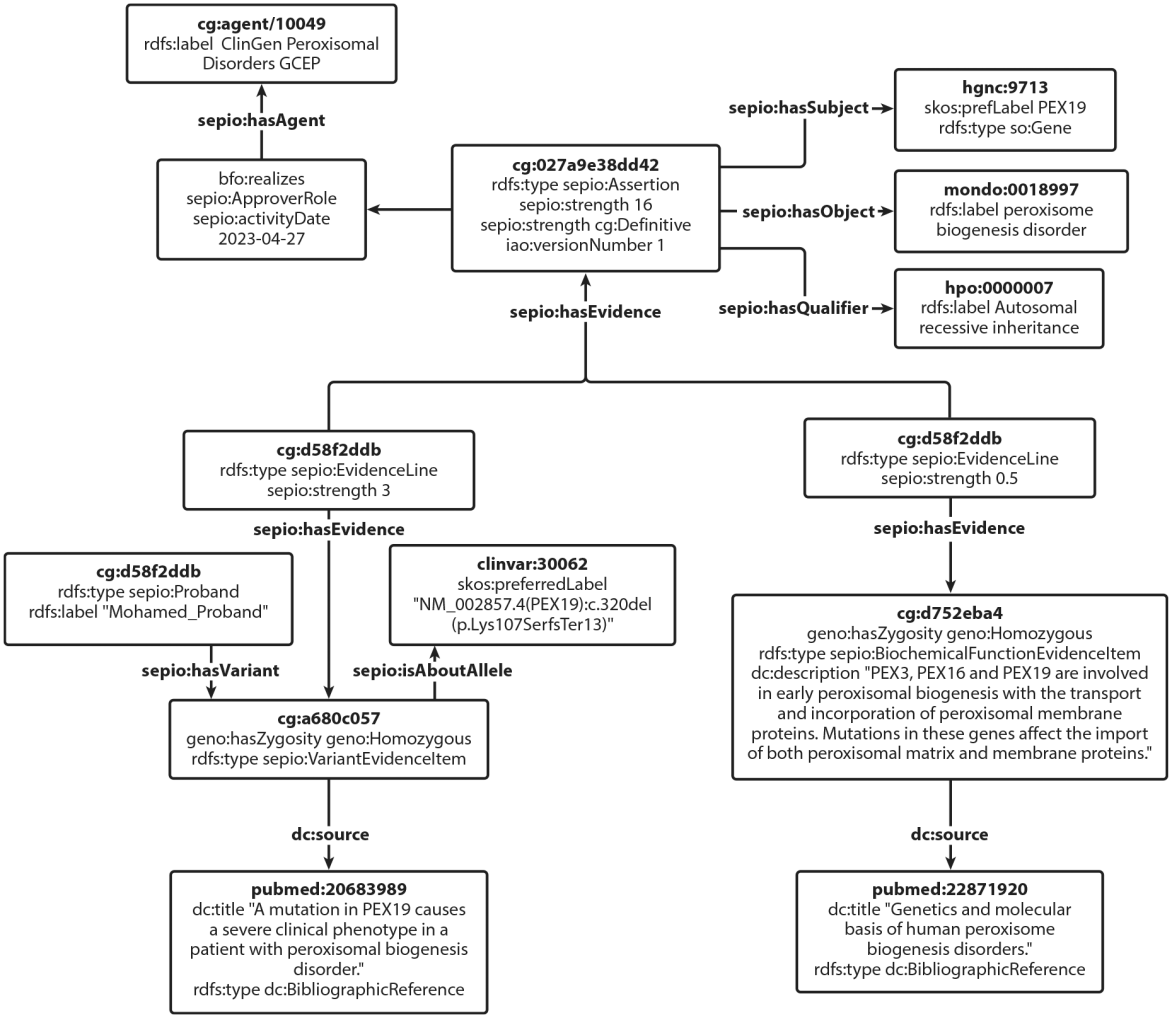
Example of data structure. When ClinGen Gene–Disease Validity curations are processed by GeneGraph, the data structure is transformed to align with the Scientific Evidence and Provenance Information Ontology data model. The Gene Curation Interface generates durable universally unique identifiers for most entities created during curation; GeneGraph leverages these and generates durable identifiers of its own so that every aspect of the curation is structured in a way that can be leveraged in many different ways by downstream systems. The structure uses an ontological foundation for data types, descriptive elements, and relationships, allowing ClinGen curations to be merged with other data by leveraging semantic web technologies. Similar concepts are represented in a consistent way; for example, the score a curator applies to a given piece of evidence has the same data model regardless of the type of evidence being scored. This truncated example of the data structure for curation scoring offers a sense of how the reasoning supporting a curation is represented using structured data. The data represented are from the same *PEX19*/peroxisome biogenesis disorder/autosomal recessive inheritance gene–disease–mode of inheritance used in Figure 3. A complete data structure can be viewed in Supplemental Figure 2.

The entire Gene–Disease Validity curation process, from conception to publication, relies on several different applications developed to support this workflow (Figure 4), primarily GeneTracker, the GCI, GeneGraph, the DEx, and the ClinGen website. These products arose based on need and are developed and maintained by different teams that work collaboratively to support the Gene–Disease Validity curation workflow. Given that systems are maintained by different teams based at different institutions, ClinGen leadership encouraged the development of an architecture that supports loose coupling of software products. The DEx is the foundation of this architecture. It is an asynchronous, event-oriented architecture backed by a deployment of Apache Kafka (36). In our environment, this offers several advantages over alternative architectures:

- Systems are less dependent on the availability of other entities to provide services to their users. In an API-based infrastructure, the structures supporting user-facing systems need to have an extremely high level of reliability to provide acceptable performance.
- Troubleshooting interactions between systems is made significantly easier. One can simply consult the log preserved by Apache Kafka to see who sent what and when. If a message was never sent, or if the expected format of a message is different than the one offered, that can be made absolutely clear to the engineers working through a problem.
- Auditability is guaranteed for processes that cross system boundaries. Since all data need to pass from our curation interfaces across the DEx to be shared with the public, all publicly shared data are effectively archived by this system.
- Ease of adding new systems or new features to existing systems is improved. Since all data shared between systems are preserved on the DEx, it is possible for systems to incorporate previously unused data simply by reading any existing stream from the beginning and listening for new messages thereafter.
- This system allows different software teams to work at different cadences. For example, the GCI was able to publish classifications before the features were built into GeneGraph and the website to receive these data.
- Systems can incorporate only the data they need from upstream systems. If more data are needed at a later date, the topic can be reprocessed for the data needed to meet the new requirements. For example, data from the DEx were used to retroactively apply versions to Gene Disease–Validity classifications.
- This architecture alleviates the complex choreography needed for schema changes in a shared-database architecture, at the cost of client systems needing to maintain their own database systems for query, as well as backward compatibility with previous message formats.

### ClinGen Website

**Figure 5:**
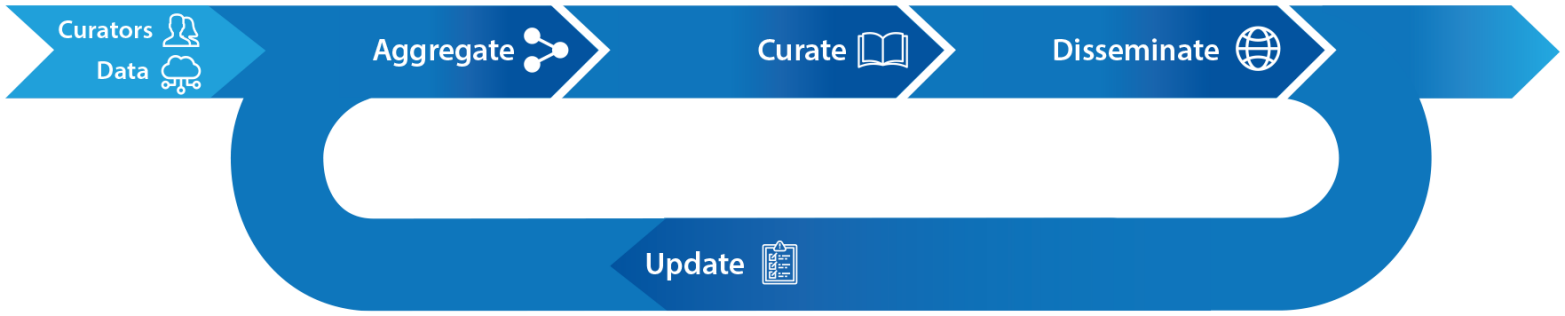
Gene–Disease Validity curation lifecycle. The Gene–Disease Validity curation lifecycle begins with biocurators (curators) collecting and annotating data (aggregate), then applying standards and frameworks (curate), with the ultimate goal of publishing records for use by the clinical community (disseminate). It is expected that many Gene–Disease Validity curations will need to be updated based on growing bodies of knowledge about the gene in relation to the disease; this is a process termed recuration. Recuration is especially necessary for gene–disease classifications that are classified as Moderate, Limited, and Disputed.

The final Gene–Disease Validity classifications are displayed for public consumption on the ClinGen website. As of January 2024, there are 56 active GCEPs that have curated 2,480 total Gene–Disease Validity classifications (based on 2,084 unique genes). While GCEPs are encouraged to publish their work in scientific journals, the living version of each classification is maintained on the ClinGen website (with updated numbers available at https://search.clinicalgenome.org/kb/gene-validity), thus allowing users to see the most up-to-date information for a curation, which will change and evolve with time (a process called recuration) (Figure 5). For those who are interested in knowing when a ClinGen Gene–Disease Validity curation has been updated, the website provides a mechanism to notify users of such information.

The ClinGen website also provides information on a GCEP’s scope of work. The scope of work, i.e., the gene list, is based on the requirements of the specific clinical domain working group and may vary in size. The scope of work and curation progress for all approved ClinGen GCEPs can be found at https://search.clinicalgenome.org/kb/affiliate. The range of work of different clinical domains published by ClinGen GCEPs includes the following:

- Cardiovascular disorders, such as arrhythmogenic right ventricular cardiomyopathy (37), dilated cardiomyopathy (38), heritable thoracic aortic aneurysm and dissection (39), hypertrophic cardiomyopathy (40), and long QT syndrome (41)
- Hearing loss (42)
- Hereditary cancers, such as breast and ovarian cancer (43), and colorectal cancer/polyposis (44)
- Inborn errors of metabolism, such as fatty acid oxidation disorders (45) and peroxisomal disorders (35)
- Mitochondrial diseases (46)
- Neurodevelopmental disorders, such as epilepsy (47) and intellectual disability/autism (48)
- Neurological disorders, such as amyotrophic lateral sclerosis (49)
- RASopathies (50)

Gene–Disease Validity classifications are presented alongside information on ClinGen’s other curation products on the website. Gene–Disease Validity precurations and classifications are discoverable by gene, disease, or expert panel. Within each curation, a rubric explaining how the scoring of evidence led to the classification is visible. On separate tabs, scoring and descriptions for individual pieces of evidence can be seen. In addition, information from precuration in GeneTracker leading to the selection of the disease term to use in curation, as well as associated terms that are considered to be included for the purposes of classification (lumping and splitting), is shown. Detailed information can be downloaded in a variety of formats from any of the evidence tables. Summary information for all Gene–Disease Validity classifications can be downloaded directly from the website as comma-separated values (https://search.clinicalgenome.org/kb/downloads#section_gene-disease-validity).

### Integrations

ClinGen Gene–Disease Validity classification data have been integrated into many downstream gene–disease related resources, including the Gene Curation Coalition (GenCC) (51), OMIM (1), the University of California Santa Cruz (UCSC) Genome Browser (52), National Center for Biotechnology Information Genome Data Viewer (53), and DECIPHER (54). For instance, the UCSC Genome Browser allows users to select the Gene–Disease Validity track within the genomic browser view in order to display ClinGen Gene–Disease Validity classifications, which are labeled with the disease term (and gene name shown via a hover-over function) and color-coded based on the strength of their classification. The GenCC integrates ClinGen classifications with assessments from several other curation sources, including commercial labs and government or nonprofit health systems. This particular integration demonstrates the significance of using standard ontologies and vocabularies, as it makes combining ClinGen data with data from other sources far more feasible.

While ClinGen members can currently request data from the GCI development team, there is not yet direct access to the full GCI data. We intend to expand the availability of the full GCI dataset to the entire internal and external communities, including through the GraphQL API mentioned above, as well as via batch and streaming downloads. This will facilitate deeper integrations with resources making use of ClinGen Gene–Disease Validity data, as well as novel uses of the data. Given the rigorous standards for ClinGen classifications as well as our extensive use of standard terminology, these data may be especially useful as a training dataset in machine learning applications based on knowledge graphs or large language models. We intend to use a format based on the emerging VA GKS specifications being developed by the GA4GH, which would support the aim to have it serve as a standard for the representation for gene–disease relationship data (55, 56). Once this is achieved, the GraphQL API will be updated to reflect these standards, and features will be added to make it suitable for public use.

## CONCLUSION AND FUTURE DIRECTIONS

The importance of an openly available, authoritative resource on the relationships between genes and human disease cannot be overstated. By supporting the curation and dissemination of information about these relationships, the ClinGen Gene–Disease Validity infrastructure serves as an essential resource for the practice of genomic medicine as well as new research in this domain. New gene discoveries are occurring frequently, as evidenced by the work of the Centers for Mendelian Genomics, which indicates a rate of ~200 new genes per year (57), so updating and adding to the Gene–Disease Validity classifications and sharing these data with the public will be essential for a complete view of the strength of evidence implicating new genes with diseases. In addition to new gene–disease discoveries, additional publications implicating an already asserted relationship will emerge that could potentially alter the current strength of evidence for a gene’s relationship with disease. To stay up to date with the growing knowledge base, the ClinGen GCWG developed recommendations for a process, termed recuration (Figure 5), to set consistent standards for the re-evaluation (including the aggregation, curation, and dissemination of new or updated data) of gene–disease relationships that do not currently meet a classification of Definitive or Refuted, largely based on an elapsed time interval from the previously approved classification (the SOP is shown in Supplemental Figure 3). The recuration process also highlights the need for versioned classifications. Informatically, curation versioning creates a time stamp of the classification approval, which is linked with the data reviewed at the time of the approval. ClinGen is currently in the process of versioning all records across Gene–Disease Validity classifications, as well as other curation activities, to allow for greater transparency to our data consumers. Versioning will also facilitate future data science initiatives and requests from either internal ClinGen users or external website consumers, including comparative analyses of classifications over time, any data that supported these changes, and the general reason why a classification was updated (e.g., new evidence, new SOP, re-evaluation of existing evidence), which hopefully will improve standards for the establishment of gene–disease relationships from the initial discovery.

In conclusion, ClinGen has developed and evolved its curation workflows and data platform over 10 years to support expert-informed Gene–Disease Validity curation and account for new standards and evidence that emerge over time. This process has allowed ClinGen to be at the forefront of publishing transparent, open-access data on Gene–Disease Validity, and in doing so to achieve its goal of being an authoritative central resource defining the clinical relevance of genes for use in precision medicine and research.

## Supplementary Material

Wright2024_Supp1

Wright2024_Supp3

Wright2024_Supp2

## Figures and Tables

**Table 1:** Ontologies and structured vocabularies

Ontology/vocabulary system	Concept covered	Reference	Resource website
Basic Formal Ontology	Supporting information for ontologies	Arp et al. (15)	https://basic-formal-ontology.org
BioAssay Ontology	Chemical biology screening assays	Visser et al. (16)	http://bioassayontology.org
Cell Ontology	Cell lines and cell culture models	Diehl et al. (17)	https://obophenotype.github.io/cell-ontology
ClinGen Allele Registry	Canonical alleles	Pawliczek et al. (18)	https://reg.clinicalgenome.org/
Dublin Core Metadata Initiative	Metadata terms	NA	https://www.dublincore.org/specifications/dublin-core
Experimental Factor Ontology	Cell lines and cell culture models	Malone et al.(19)	https://www.ebi.ac.uk/efo
Gene Ontology	Cell processes and interactions	Aleksander et al. (20)	https://geneontology.org
GENO Ontology	Genotypes and their association with phenotypes	NA	https://github.com/monarch-initiative/GENO-ontology
HUGO Gene Nomenclature Committee	Gene nomenclature	Seal et al. (21)	https://www.genenames.org
Human Genome Variation Society	Variant nomenclature	den Dunnen et al. (22)	https://hgvs-nomenclature.org/
Human Phenotype Ontology	Phenotypes and modes of inheritance	Kohler et al. (9)	https://hpo.jax.org
Mammalian Phenotype Ontology	Nonhuman model organisms	Smith & Eppig (23)	https://www.informatics.jax.org/vocab/mp_ontology
Measurement Method Ontology	Clinical and phenotypic measurement methods	Smith et al. (24)	https://obofoundry.org/ontology/mmo.html
Molecular Interactions Controlled Vocabulary	Protein-protein interactions	NA	https://github.com/HUPO-PSI/psi-mi-CV
Monarch Disease Ontology	Diseases	Visser et al. (16)	https://mondo.monarchinitiative.org
NCBI Organismal Taxonomy	Organisms	NA	https://www.ncbi.nlm.nih.gov/taxonomy
Online Mendelian Inheritance in Man	Diseases and phenotypes	Amberger et al. (1)	https://omim.org
Relation Ontology	Relationships between ontologies	Smith et al. (25)	https://obofoundry.org/ontology/ro.html
Scientific Evidence and Provenance Information Ontology	Provenance of scientific claims	Brush et al. (26)	https://github.com/monarch-initiative/SEPIO-ontology
Sequence Ontology	Molecular consequences	Eilbeck et al. (27)	http://www.sequenceontology.org
Simple Knowledge Organization System	Sharing and linking knowledge data model	NA	https://www.w3.org/TR/skos-reference
Uber-Anatomy Ontology	Organs and tissues	Mungall et al. (28)	https://obophenotype.github.io/uberon

Abbreviation: NA, not applicable.

## Abbreviations

Gene Curation Working Group (GCWG): develops evidence-based methods for evaluating gene–disease relationships to support gene curation activities across the ClinGen project
Gene Curation Expert Panel (GCEP): multi-institutional team with broad expertise in a particular field or condition that is approved by ClinGen to curate Gene–Disease Validity classifications
Lumping and Splitting Working Group (LSWG): provides guidance for biocurators and experts in delineating the most appropriate disease entity(ies) for classification
GeneTracker: a ClinGen software application that assists GCEPs in managing their evidence and conclusions regarding the lumping and splitting of disease phenotypes
Gene Curation Interface (GCI): a ClinGen software application designed to facilitate the curation of gene–disease relationships by curators working as part of ClinGen GCEPs
Human Genome Variation Society (HGVS): an international organization focused on standardizing the description of human sequence variations
ClinGen Allele Registry: provides unique canonical identifiers for genetic variants
Matched Annotation from NCBI and EMBL-EBI (MANE): a collaborative project between the NCBI and EMBL-EBI that aims to define a genome-wide set of representative transcripts and corresponding proteins for human protein-coding genes
Apache Kafka: an open-source distributed event streaming platform designed for handling real-time data feeds
Data Exchange (DEx): ClinGen platform, data models, and tools that enable an environment of standardized exchange of the full suite of genomic knowledge curated by ClinGen
GeneGraph: a bioinformatics tool developed to normalize the representation of curated Gene–Disease Validity data and facilitate use of the data by downstream resources
Apache Jena: an open-source Java framework for building semantic web and linked data applications; it provides an API to extract and/or write data to Resource Description Framework graphs
Scientific Evidence and Provenance Information Ontology (SEPIO): ontological framework developed to support the description of evidence and provenance information for scientific claims
Global Alliance for Genomics and Health (GA4GH): harmonizes health-related data via the development of standards, frameworks, and policies across the global genomics community
Genomic Knowledge Standards (GKS): works to define a common computational language for GA4GH to describe variants to facilitate the exchange of genomic knowledge through common APIs, schemas, and software
Clinical domain working group: team of experts in a given clinical domain working to evaluate the clinical validity of gene–disease relationships and pathogenicity of individual genetic variants
