# Supplementary material for: Generating Clinical-Grade Gene–Disease Validity Classifications Through the ClinGen Data Platforms
Source: Annu Rev Biomed Data Sci. Author manuscript; Available in PMC 2025 Aug 1. (PMC12001867; doi:10.1146/annurev-biodatasci-102423-112456)
Supplement: Wright2024_Supp1 [file NIHMS2073507-supplement-Wright2024_Supp1.pdf]

Downloaded from www.annualreviews.org. Guest (guest) IP: 108.6.200.146 On: Wed, 09 Apr 2025 20:28:05

S1: Supplementary File 1 - An example of a complete Curation Summary Table from the GCI.

This table summarizes every individual piece of scored evidence captured as part of the ClinGen Gene-Disease Validity classification fo the PEX19 (HGNC:9713)/peroxisome biogenesis disorder (MONDO:0019234)/Autosomal recessive inheritance (HP:0000007) GDM within the GCI, as curated by the ClinGen Peroxisomal Disorders GCEP (Mohan et al., 2023; PMID: 37236006). A version of this matri table is replicated in the final classification published to the ClinGen website: https://search.clinicalgenome.org/kb/genes/HGNC:9713

✖ Close

Evidence Summary

PEX19 – peroxisome biogenesis disorder – *Autosomal recessive inheritance*

Classification owner: Peroxisomal Disorders
Calculated classification: Definitive
Modified classification: No Modification
Reason for modified classification: Unpublished data on individual with PEX19 variant contributes 0.5 points to the genetic evidence. This information can be added to the curation once the data is published.
SOP: Gene Clinical Validity Standard Operating Procedures (SOP), Version 9

Classification status: APPROVED PUBLISHED
Date classification saved: 2023 Apr 27, 3:21 pm
Final Approval Date: 2023 Apr 27
Date classification published: 2023 Apr 27, 3:22 pm
Replication Over Time: Yes

Contradictory Evidence? Proband: **No**, Case-Control: **No**, Experimental: **No**
Disease: peroxisome biogenesis disorder

Evidence Summary

The relationship between PEX19 and peroxisome biogenesis disorder (type 12A (Zellweger) included), an autosomal recessive disorder, was evaluated using the ClinGen Clinical Validity Framework as of January, 2020. PEX19 encodes a peroxisome membrane protein involved in early peroxisome membrane biosynthesis prior to matrix protein import. Peroxisomal biogenesis disorders are caused by defects in various stages of peroxisomal protein import and/or peroxisome biogenesis, involving at least 14 PEX genes. PEX19 belongs to complementation group J and mutations in the gene result in cells devoid of peroxisomal remnants (Waterham and Ebberink 2012, PMID 22871920) or with a small number of enlarged peroxisomes (Ebberink et al, 2010, PMID 20647552).

PEX19 was first reported in relation to autosomal recessive Peroxisome biogenesis disorder in 1999. (Matsuzono et al, PMID: 10051604). At least 3 nonsense or frameshift variants have been reported in humans. Evidence supporting this gene-disease relationship includes case-level data and experimental data.

Summary of Case Level Data (12 points): Variants in this gene have been reported in at least 5 probands in 5 publications (PMID: 10051604, 20683989, 21031596, 30561787, 36931687). Variants in this gene segregated with 4 additional family members. The mechanism for disease is expected to be homozygous loss of function.

Summary of experimental data (4 points): This gene-disease association is supported by in vitro functional assays (PMIDs 21669930, 28526747, 12096124, 10051604). PEX19 interacts with PEX3, PEX11B and PEX16.

In summary, the PEX19-peroxisome biogenesis disorder gene-disease relationship is definitive. This has been repeatedly demonstrated in both the research and clinical diagnostic settings, and has been upheld over time. This classification was approved by the ClinGen Peroxisomal Disorders GCEP on January 17, 2020 (SOP Version 7). This gene-disease pair was originally evaluated by the Peroxisomal Disorders on January 17, 2020. It was reevaluated on April 27, 2023. As a result of this reevaluation, the classification did not change. The genetic evidence was updated and scored according SOP v9 and a new case report added, which maxed out the genetic evidence score.

Calculated Classification Matrix

Evidence Type	Variant Count	Proband Count	Total Points	Points Counted

Genetic Evidence	Case-Level	Variant	**Autosomal Dominant OR X-linked Disorder**	Predicted or proven null variant		0	0	0	0
				Other variant type		0	0	0	
			**Autosomal Recessive Disorder**	Predicted or proven null variant		10	6	13.2	12
				Other variant type		2			
		**Segregation**			**Summed LOD**	**Family Count**		1	1
				Candidate gene sequencing	3.16	1			
				Exome/genome or all genes sequenced in linkage region	0	0			
				**Total Summed LOD Score**	**3.16**				
	**Case-Control**					**Case-Control Cohort**			
						0		0	0
	**Genetic Evidence Total**								**12**
Experimental Evidence	**Functional**			Biochemical Functions			2	1	2
				Protein Interactions			1	2	
				Expression			0	0	
	**Functional Alteration**			Patient cells			0	0	1
				Non-patient cells			1	1	
	**Models**			Non-human model organism			0	0	1
				Cell culture model			0	0	
	**Rescue**			Rescue in human			0	0	
				Rescue in non-human model organism			0	0	
				Rescue in cell culture model			0	0	
				Rescue in patient cells			1	1	
	**Experimental Evidence Total**								**4**
**Total Points**									**16**

Scored Genetic Evidence: Case Level (variants)

Proband Label	Variant Type	Variant	Reference (PMID)	Proband Sex	Proband Age	Proband Ethnicity	Proband Phenotypes	Proband Previous Testing	Proband Methods of Detection	Functional Data (Explanation)	De Novo (paternity/ maternity confirmed)	Score Status	Proband Points (default points)	Proband Counted Points	Explanation
Mohamed_Proband	Predicted or proven null	NM_002857.4 (PEX19):c.320del (p.Lys107SerfsTer13)	Mohamed S, et al., **2010**, PMID: 20683989	Female	**Age of Death:** 16 Months		**HPO term(s):**<br>• Poor suck<br>• Cranial asymmetry<br>• Scaphocephaly<br>• Triangular face<br>• Abnormality of the hairline<br>• Asymmetry of the ears<br>• Wide anterior fontanel<br>• Epicanthus<br>• Periorbital fullness<br>• Prominence of the premaxilla<br>• Prominent nose<br>• Wide nasal bridge<br>• Short chin<br>• Retrognathia<br>• Infantile muscular hypotonia<br>• Lethargy<br>• Weak cry<br>• Hyperactive deep tendon reflexes<br>• Conjugated hyperbilirubinemia<br>• Unconjugated hyperbilirubinemia<br>• Elevated hepatic transaminase<br>• Diffuse demyelination of the cerebral white matter<br>• Cerebral atrophy<br>• Patent ductus arteriosus<br>• Atrial septal defect<br>• Very long chain fatty acid accumulation<br><br>**Free text:**<br>Proband had an Apgar score of 6 and 8 at 1 and 5 min. Birth weight = 2.58 kg. She was noted to be inactive on the first day of life. She also had widely open posterior fontanele, absent Moro reflex, inconsistent rooting, absent	Immunoflorescence microscopy with antibody against catalase in patient fibroblasts showed absence of peroxisomes. Complementation studies showed that the gene affected was PEX19.	**Description of genotyping method:** The authors mention that mutation analysis of the PEX19 gene was performed.	No	No	Score	**3** (3)	3.00	The proband was homozygous for a single-base-pair deletion that causes a frameshift and subsequent early termination. The resulting mRNA is expected to undergo NMD. The evidence is awarded default points.

Proband Label	Variant Type	Variant	Reference (PMID)	Proband Sex	Proband Age	Proband Ethnicity	Proband Phenotypes	Proband Previous Testing	Proband Methods of Detection	Functional Data (Explanation)	De Novo (paternity/ maternity confirmed)	Score Status	Proband Points (default points)	Proband Counted Points	Explanation
							palmar grasp. Her weight, length, and head circumference were at the 3rd centile. Systolic murmur was noted at the left sternal border. Tests for ammonia, lactate, TORCH screen, chromosomes, serum amino acids, acylcarnitine, and urine organic acids were normal. CT scan of the brain showed diffuse bilateral changes involving the frontotemporal and parieto-occipital regions, and small bilateral intraventricular hemorrhage in the posterior horns of the lateral ventricles. EEG showed diffuse disturbance of cerebral activity dysynchronization and depression with a sharp transient wave over the central and temporal region. Patient fibroblasts showed deficient activity of DHAPAT, abnormal VLCFA and pristanic acid β-oxidation, deficient phytanic acid α-oxidation and abnormally processed acyl-CoA oxidase and peroxisomal thiolase.<br><br>Plasma peroxisomal functions:<br><br>C22:0 = 20.8 µmol/L (control: 21–103 µmol/L); C24:0 = 48.2 µmol/L (control: 22–87 µmol/L); C26:0 = 9.72 µmol/L (control: 0.05–1.97 µmol/L); C24:0/C22:0 = 3.32 (control: 0–1.15); C26:0/C22:0 = 0.467 (control: 0–0.028); Phytanic acid = 1.6 (control: 0–10								

Proband Label	Variant Type	Variant	Reference (PMID)	Proband Sex	Proband Age	Proband Ethnicity	Proband Phenotypes	Proband Previous Testing	Proband Methods of Detection	Functional Data (Explanation)	De Novo (paternity/ maternity confirmed)	Score Status	Proband Points (default points)	Proband Counted Points	Explanation
							µmol/L); Pristanic acid = 0.31 (control: 0-1 µmol/L)								
Matsuzono_PBDJ-01	Predicted or proven null	NM_002857.4 (PEX19):c.763 dup (p.Met255Asn fsTer25)	Matsuzono Y, et al., **1999**, PMID: 10051604	Unknown			**Free text:** Skin fibroblast cell lines from patient with PBD, belonging to complementation group J		**Description of genotyping method:** RT-PCR was performed on cDNA from patient fibroblasts. Ten clones were sequenced and found to have the same variant. Sequencing of genomic DNA also gave the same result.	Yes (The authors note that back transfection of the variant cDNA to patient fibroblasts as well as PEX-19 CHO-cell mutant did not restore peroxisome biosynthesis, indicating that the variant is inactivating.)	No	Score	**4** (4)	3	The proband was homozygous for 1-bp duplication towards the end of exon 6. The variant causes a frameshift and introduction of 28 distinct amino acids and termination at position 283. The variant is reported at a frequency of 0.0002787 (7 / 25118 Finnish alleles), with 0 homozygotes. The overall frequency is 0.00003541. Note, the variant is reported in the paper as 764insA. Per HGVS, this variant is c.763dup.

Proband Label	Variant Type	Variant	Reference (PMID)	Proband Sex	Proband Age	Proband Ethnicity	Proband Phenotypes	Proband Previous Testing	Proband Methods of Detection	Functional Data (Explanation)	De Novo (paternity/ maternity confirmed)	Score Status	Proband Points (default points)	Proband Counted Points	Explanation
Ebberink_PEX19 Proband 1	Predicted or proven null	NM_002857.4 (PEX19):c.320 del (p.Lys107Serf sTer13)	Ebberink MS, et al., **2011**, PMID: 21031596	Unknown			**Free text:** Primary skin fibroblast cell line from an individual diagnosed with Zellweger syndrome was used for mutation analysis. Individuals were diagnosed based on: (1) metabolite analysis in plasma of very long chain fatty acids (VLCFAs), bile acid intermediates, phytanic acid, pristanic acid, pipecolic acid and plasmalogens and/or (2) detailed studies in fibroblasts, including VLCFA analysis, C26:0 and pristanic acid β-oxidation, phytanic acid α-oxidation, and dihydroxyacetonephosphate acyltransferase (DHAPAT) activity analysis and (3) absence of peroxisomes assessed by on catalase immunofluorescence (IF) microscopy	PEX cDNA transfection assay was used for genetic complementation studies. Transfection with PEX3, PEX16 and PEX19 was performed when patient fibroblasts showed no peroxisomal membrane remnants based on immunofluorescence microscopy with antibodies against ALDP, a peroxisomal membrane protein. After assignment of genetic complementation groups, the respective gene was analyzed.	**Method 1:** Sanger sequencing **Description of genotyping method:** PEX19 gene was analyzed by sequencing all exons plus flanking intronic sequences amplified from gDNA from primary skin fibroblast cultures.	No	No	Score	**0** (3)	0.00	The 1-bp deletion in exon 3 was identified in the PEX19 gene in primary skin fibroblast cell line from a patient diagnosed with Zellweger syndrome. The variant is expected to cause a frameshift and premature termination of translation at amino acid position 119. The resulting transcript is predicted to undergo NMD. The proband is assumed homozygous. The variant is not found in gnomAD. The proband is not scored any points as the same variant has been scored in Mohamed et al, 2010, PMID: 20683989. As only patient fibroblasts have been used in this paper by Ebberink et al, it is unclear if the sample is from the same proband.

Proband Label	Variant Type	Variant	Reference (PMID)	Proband Sex	Proband Age	Proband Ethnicity	Proband Phenotypes	Proband Previous Testing	Proband Methods of Detection	Functional Data (Explanation)	De Novo (paternity/ maternity confirmed)	Score Status	Proband Points (default points)	Proband Counted Points	Explanation
Ebberink_PEX19 Proband 2	Predicted or proven null	NM_002857.4 (PEX19):c.769 C>T (p.Gln257Ter)	Ebberink MS, et al., **2011**, PMID: 21031596	Unknown			**Free text:** Primary skin fibroblast cell line from an individual diagnosed with Zellweger syndrome was used for mutation analysis. Individuals were diagnosed based on: (1) metabolite analysis in plasma of very long chain fatty acids (VLCFAs), bile acid intermediates, phytanic acid, pristanic acid, pipecolic acid and plasmalogens and/or (2) detailed studies in fibroblasts, including VLCFA analysis, C26:0 and pristanic acid β-oxidation, phytanic acid α-oxidation, and dihydroxyacetonephosphate acyltransferase (DHAPAT) activity analysis and (3) absence of peroxisomes assessed by on catalase immunofluorescence (IF) microscopy	PEX cDNA transfection assay was used for genetic complementation studies. Transfection with PEX3, PEX16 and PEX19 was performed when patient fibroblasts showed no peroxisomal membrane remnants based on immunofluorescence microscopy with antibodies against ALDP, a peroxisomal membrane protein. After assignment of genetic complementation groups, the respective gene was analyzed.	**Method 1:** Sanger sequencing	No	No	Score	**3** (3)	3.00	The substitution in exon 6 was identified in the PEX19 gene in primary skin fibroblast cell line from a patient diagnosed with Zellweger syndrome. The nonsense variant causes premature termination of translation and the resulting transcript is predicted to undergo NMD. The proband is assumed homozygous. The variant is not found in gnomAD. The paper reports the variant in Table 11 as c.739C>T, and the amino acid consequence as Q257X. However, the correct nucleotide position for the nonsense variant is c.769C>T. This was also confirmed by the authors when ClinGen Peroxisomal Disorders GCEP reached out to them.

Proband Label	Variant Type	Variant	Reference (PMID)	Proband Sex	Proband Age	Proband Ethnicity	Proband Phenotypes	Proband Previous Testing	Proband Methods of Detection	Functional Data (Explanation)	De Novo (paternity/ maternity confirmed)	Score Status	Proband Points (default points)	Proband Counted Points	Explanation
Alshenaifi_16DG1051	Other Variant Type	NM_002857.4 (PEX19):c.161 C>T (p.Ser54Leu)	Alshenaifi J, et al., **2019**, PMID: 30561787	Female	**Age of Report:** 29 Years		**HPO term(s):** • Generalized hypotonia<br>• Seizure<br>• Global developmental delay<br>• Absent speech<br>• Very long chain fatty acid accumulation<br>• Febrile seizure (within the age range of 3 months to 6 years)<br>• Spasticity<br>• Hyperreflexia<br>• Short stature<br>**Free text:** Proband had a stiff gait. She started to walk at 4y and had few words. Seizures continue and are difficult to control, which are exacerbated during her menstrual cycle. Her VLCFA levels were mildly elevated while levels were normal in all her 4 affected siblings.	Plasma VLCFA, pristanic, and phytanic acid levels were measured	**Method 1:** Next generation sequencing panels; **Method 2:** Sanger sequencing **Description of genotyping method:** NGS panels in combination with autozygome analysis was employed. WES was used in cases where a mutation was not detected. Variants were confirmed by Sanger.	No	No	Score	**0.2** (0.2)	0.20	The proband was homozygous for the missense change, Ser54Leu. The variant segregated in the five affected siblings in this family. The variant is absent in gnomAD. In-silico predictions do not predict a damaging effect. In the absence of functional evidence, the proband is scored minimal points.

Proband Label	Variant Type	Variant	Reference (PMID)	Proband Sex	Proband Age	Proband Ethnicity	Proband Phenotypes	Proband Previous Testing	Proband Methods of Detection	Functional Data (Explanation)	De Novo (paternity/ maternity confirmed)	Score Status	Proband Points (default points)	Proband Counted Points	Explanation
Adiyapatham_Proband	Predicted or proven null	NM_002857.4 (PEX19):c.281 T>A (p.Leu94Ter)	Adiyapatham S, et al., **2023**, PMID: 36931687	Male	**Age of Death:** 8 Days		**HPO term(s):**<br>• Wide nasal bridge<br>• Wide anterior fontanel<br>• Prominent metopic ridge<br>• Low-set ears<br>• Hypertelorism<br>• Large posterior fontanelle<br>• Retrognathia<br>• Micrognathia<br>• Abnormal pinna morphology<br>• Webbed neck<br>• Single transverse palmar crease<br>• Cryptorchidism<br>• Corneal opacity<br>• Hypotonia<br>• Poor suck<br>• Hypospadias<br>• Weak cry<br>• Systolic heart murmur<br>• Bilateral talipes equinovarus<br>**Free text:**<br>Proband was in NICU after birth due to antenatal detection of congenital anomalies and required mechanical ventilation due to recurrent apnea. Antenatal scans suggested intracranial translucency with hypoplastic inferior cerebellar vermis. He had poor Moro's and rooting reflexes.		**Method 1:** Exome sequencing	No	No	Score	**3** (3)	3.00	The proband was homozygous for the nonsense variant, Leu94Ter, in exon 3/8, and NMD is predicted. Third-degree consanguinity is reported in the family. Proband had a similarly affected elder sibling who also passed away in the neonatal stage, without molecular testing having been performed.

Total Variant points: 12.20

Scored Genetic Evidence: Case Level (segregation)

Family (Proband) Label	Reference (PMID)	Family Ethnicity	Family Phenotypes	Family MOI	# Aff	# Unaff	LOD Score	LOD Score Counted	Sequencing Method
Alshenaifi_PEX19 family (Alshenaifi_16DG1051)	Alshenaifi J, et al., **2019**, PMID: 30561787		**HPO term(s):**<br>• Generalized hypotonia<br>• Seizure<br>• Global developmental delay<br>• Absent speech		5	6	**Calculated:** 3.16	Yes	Candidate gene sequencing

Genetic Evidence: Case Level (family segregation information without proband data or scored proband data)

No segregation evidence for a Family without a proband was found.

Genetic Evidence: Case-Control

No scored Case-Control evidence was found.

Experimental Evidence

Label	Experimental Category	Reference	Explanation	Score Status	Points (default points)	Explanation
Waterham_PEX function	**Biochemical Function** A	Waterham HR, et al., **2012**, PMID: 22871920	PEX3, PEX16 and PEX19 are involved in early peroxisomal biogenesis with the transport and incorporation of peroxisomal membrane proteins. Mutations in these genes affect the import of both peroxisomal matrix and membrane proteins.	Score	**0.5** (0.5)	
Fransen_PEX19 interactions	**Protein Interactions** physical association (MI:0915)	Fransen M, et al., **2002**, PMID: 12096124	A non-transcription-based bacterial 2-hybrid system, which exploits the cAMP signaling cascade in E. coli cya cells, deficient in endogenous adenylate cyclase, was used. Matrix experiments were performed with the 12 full-length peroxins, tested systematically for pairwise interactions. PEX19 was found to interact with PEX3, PEX11B, PEX13 and PEX16.	Score	**2** (0.5)	The evidence is scored increased points for interaction with 4 other PEX genes.
Mast_S2 cells function	**Biochemical Function** B	Mast FD, et al., **2011**, PMID: 21669930	In patients with mild forms of PBDs, a small number of import-competent, enlarged peroxisomes are observed (from PMID: 20647552)	Score	**0.5** (0.5)	
Matsuzono_Rescue in patient cells	**Rescue** Patient cells	Matsuzono Y, et al., **1999**, PMID: 10051604	Cells stained with antibodies to catalase showed positive staining, demonstrating rescue of peroxisome formation. This was also seen in CHO mutants.	Score	**1** (1)	

Label	Experimental Category	Reference	Explanation	Score Status	Points (default points)	Explanation
Agrawal_FA	**Functional Alteration** Non-patient cells	Agrawal G, et al., **2017**, PMID: 28526747	Yeast cells expressing wild-type Pex19 showed normal peroxisome size. Complete deletion of Pex19 in cells resulted in no growth. Cells with the deletion mutants showed absence of or slow growth. Cells that survived did not contain peroxisome or had enlarged peroxisomes.	Score	**1** (0.5)	The evidence is awarded increased points as it shows that loss of N-terminal and C-terminal regions of the gene can impact peroxisome biogenesis.
						**Total points: 5.00**

Biochemical Function: The gene product performs a biochemical function shared with other known genes in the disease of interest (A), OR the gene product is consistent with the observed phenotype(s) (B)

Protein Interactions: The gene product interacts with proteins previously implicated (genetically or biochemically) in the disease of interest

Expression: The gene is expressed in tissues relevant to the disease of interest (A), OR the gene is altered in expression in patients who have the disease (B)

Functional Alteration of gene/gene product: The gene and/or gene product function is demonstrably altered in cultured patient or non-patient cells carrying candidate variant(s)

Model Systems: Non-human model organism OR cell culture model with a similarly disrupted copy of the affected gene shows a phenotype consistent with human disease state

Rescue: The phenotype in humans, non-human model organisms, cell culture models, or patient cells can be rescued by exogenous wild-type gene or gene product

Non-scorable Evidence

No non-scorable evidence was found.

For best printing, choose "Landscape" for layout, 50% for Scale, "Minimum" for Margins, and select "Background graphics".

Close Print PDF
