# Supplementary material for: Generating Clinical-Grade Gene–Disease Validity Classifications Through the ClinGen Data Platforms
Source: Annu Rev Biomed Data Sci. Author manuscript; Available in PMC 2025 Aug 1. (PMC12001867; doi:10.1146/annurev-biodatasci-102423-112456)
Supplement: Wright2024_Supp3 [file NIHMS2073507-supplement-Wright2024_Supp3.pdf]

Downloaded from www.annualreviews.org. Guest (guest) IP: 108.6.200.146 On: Wed, 09 Apr 2025 20:29:14

S3: Supplementary File 3 - The Standard Gene-Disease Relationship Recuration Procedure(s) Version from September 2023. This is updated over time; the current version of this can be found here: https://clinicalgenome.org/site/assets/files/2164/clingen_standard_gene-disease_validity_recuration_procedures_v1.pdf

Downloaded from www.annualreviews.org. Guest (guest) IP: 108.6.200.146 On: Wed, 09 Apr 2025 20:29:14

Standard Gene-Disease Relationship Recuration Procedure(s):

This document outlines standard procedures for routine recuration of gene-disease validity for ClinGen GCEPs. All current and future GCEPs will be expected to follow these procedures. GCEPs that have completed work prior to these guidelines, but are not actively meeting, will be contacted separately to enact a plan for recuration according to the same timeline.

<u>**General recuration rules:**</u>

- Recuration of gene-disease relationships will be performed according the most current version of the Gene Disease Validity Classification SOP and the Lumping and Splitting Guidelines.
- The status and evidence for the gene-disease recuration will be updated in the Gene Tracking System.
- All recuration evidence will be entered into the current release of the Gene Curation Interface (GCI).

<u>**Standard Recuration Procedure:**</u>
All GCEPs are expected to appoint a contact person (liaison) for all ClinGen correspondence.

- Ideally, each GCEP will have at least one GCEP liaison appointed to monitor for recuration updates and communicate with their overarching Clinical Domain Working Group (CDWG) and/or the ClinGen General Gene Curation Expert Panel (GGCEP).
- GCEPs should plan to assemble annually to review previous classifications and review any new evidence.
- If the GCEP is unable to reconvene at least one biocurator and two experts from the original GCEP, the GCEP liaison will contact the CDWG to inform them so that a plan for recuration can be made.
    - In this situation, the CDWG may form an overarching GCEP for their clinical domain that may assume responsibility for the recuration and appoint the appropriate individuals to help in the recuration process, either at the biocuration level, expert level, or both.
    - Alternatively, the GGCEP may be contacted to assist with the efforts, either with help in the biocuration or in the approval processes.

Standard Gene-Disease Clinical Validity Recuration Procedure		
**Classification**	**Time for evaluation***	**Specifications for recuration**
Definitive	No set requirement	- New contradictory information is published<br>- A new disease entity assertion is made (if it falls under the purview of the group)<br>- A request for re-evaluation is made by the community
Strong	3 years from the original discovery publication date	- Consider if any contradictory evidence has been published since the last approved classification.<br>- Check if any new disease entities have been asserted.<br>- Update with any new pertinent information.
Moderate	2 years after the last approval date	- Consider if any contradictory evidence has been published since the last approved classification.<br>- Check if any new disease entities have been asserted.<br>- Update with any new pertinent information.
Limited	3 years after the last approval date	- Consider if any contradictory evidence has been published since the last approved classification.<br>- Check if any new disease entities have been asserted.<br>- Update with any new pertinent information.
No Evidence (genetic)	No set requirement	- Consider if any new evidence for the gene within your GCEP disease scope has been published since the last approved classification.<br>- Genes in this category may have had new disease entities asserted that fall outside the original GCEPs purview, in this case, the

		curations will be evaluated and performed by the ClinGen affiliated biocuration cores.
Disputed	3 years after the last approval date	- Consider if any new supportive evidence has been published<br>- Check if any new disease entities have been asserted.<br>- Update with any new pertinent information.
Refuted	No set requirement	*"Refuted" gene-disease relationships are thought to have overwhelming evidence against the asserted association, however consider:*<br>- If new supporting evidence of the gene-disease relationship is published, re-evaluation may be appropriate.<br>- A request for re-evaluation is made by the community.<br>- A new disease entity assertion is made and falls within the purview of the original group.

* These are maximum times. Ideally GCEPs would stay abreast of current literature. This can be down manually or through the use of automated electronic literature alerts from sites such as PubMed/NCBI, Cold Spring Harbor LibGuides, or F1000Prime, to decide if a gene should be prompted for earlier review.

Downloaded from www.annualreviews.org. Guest (guest) IP: 108.6.200.146 On: Wed, 09 Apr 2025 20:29:14
