# Supplementary material for: Generating Clinical-Grade Gene–Disease Validity Classifications Through the ClinGen Data Platforms
Source: Annu Rev Biomed Data Sci. Author manuscript; Available in PMC 2025 Aug 1. (PMC12001867; doi:10.1146/annurev-biodatasci-102423-112456)

S2: Supplementary File 2 - A complete data model structure for a GDM.

When ClinGen Gene-Disease Validity curations are processed by GeneGraph, the data structure is transformed to align with the SEPIO data model. The GCI generates durable identifiers (UUIDs) for most entities created during curation; GeneGraph leverages these and generates durable identifiers of its own so that every aspect of the curation is structured in a way that can be leveraged in many different ways by downstream systems. The structure uses an ontological foundation for data types, descriptive elements, and relationships, allowing ClinGen curations to be merged with other data by leveraging semantic web technologies. Similar concepts are represented in a consistent way, for example the score a curator applies to a given piece of evidence has the same data model irregardless of the type of evidence being scored. The data represented here is from MRAS (HGNC:7227)/Noonan syndrome (MONDO:0018997)/Autosomal dominant inheritance (HP:0000006) curated by the CinGen RASopathy Gene Curation Expert Panel (Grant et al., 2018; PMID: 30311384). The final classification is published to the ClinGen website: https://search.clinicalgenome.org/kb/genes/HGNC:7227

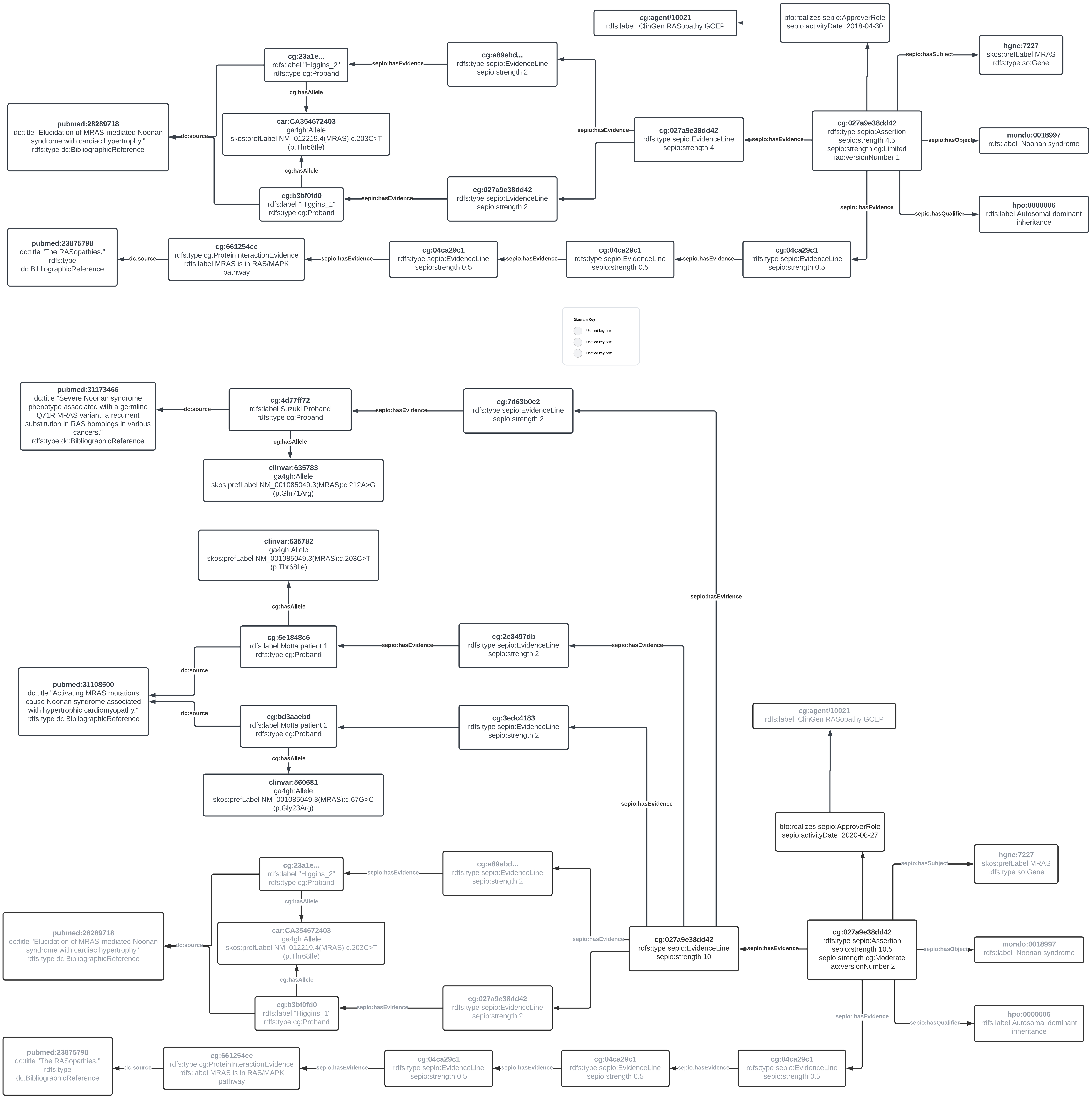

Supplement: Wright2024_Supp2 [file NIHMS2073507-supplement-Wright2024_Supp2.pdf]
